# Integrating Clinical Decision Support Into Electronic Health Record Systems Using a Novel Platform (EvidencePoint): Developmental Study

**DOI:** 10.2196/44065

**Published:** 2023-10-19

**Authors:** Jeffrey Solomon, Katherine Dauber-Decker, Safiya Richardson, Sera Levy, Sundas Khan, Benjamin Coleman, Rupert Persaud, John Chelico, D'Arcy King, Alex Spyropoulos, Thomas McGinn

**Affiliations:** 1 Institute of Health System Science Feinstein Institutes for Medical Research Manhasset, NY United States; 2 Department of Population Health New York University Grossman School of Medicine New York, NY United States; 3 Department of Psychiatry Heersink School of Medicine University of Alabama at Birmingham Medicine Birmingham, AL United States; 4 Department of Medicine Baylor College of Medicine Houston, TX United States; 5 Center for Innovations in Quality, Effectiveness, and Safety Michael E DeBakey Veterans Affairs Medical Center Houston, TX United States; 6 Physician Enterprise CommonSpirit Health Chicago, IL United States; 7 School of Psychology Fielding Graduate University Santa Barbara, CA United States; 8 Department of Medicine Donald and Barbara Zucker School of Medicine at Hofstra/Northwell Hempstead, NY United States

**Keywords:** clinical decision support system, cloud based, decision support, development, EHR, electronic health record, evidence-based medicine, health information technology, platform, user-centered design

## Abstract

**Background:**

Through our work, we have demonstrated how clinical decision support (CDS) tools integrated into the electronic health record (EHR) assist providers in adopting evidence-based practices. This requires confronting technical challenges that result from relying on the EHR as the foundation for tool development; for example, the individual CDS tools need to be built independently for each different EHR.

**Objective:**

The objective of our research was to build and implement an EHR-agnostic platform for integrating CDS tools, which would remove the technical constraints inherent in relying on the EHR as the foundation and enable a single set of CDS tools that can work with any EHR.

**Methods:**

We developed EvidencePoint, a novel, cloud-based, EHR-agnostic CDS platform, and we will describe the development of EvidencePoint and the deployment of its initial CDS tools, which include EHR-integrated applications for clinical use cases such as prediction of hospitalization survival for patients with COVID-19, venous thromboembolism prophylaxis, and pulmonary embolism diagnosis.

**Results:**

The results below highlight the adoption of the CDS tools, the International Medical Prevention Registry on Venous Thromboembolism-D-Dimer, the Wells’ criteria, and the Northwell COVID-19 Survival (NOCOS), following development, usability testing, and implementation. The International Medical Prevention Registry on Venous Thromboembolism-D-Dimer CDS was used in 5249 patients at the 2 clinical intervention sites. The intervention group tool adoption was 77.8% (4083/5249 possible uses). For the NOCOS tool, which was designed to assist with triaging patients with COVID-19 for hospital admission in the event of constrained hospital resources, the worst-case resourcing scenario never materialized and triaging was never required. As a result, the NOCOS tool was not frequently used, though the EvidencePoint platform’s flexibility and customizability enabled the tool to be developed and deployed rapidly under the emergency conditions of the pandemic. Adoption rates for the Wells’ criteria tool will be reported in a future publication.

**Conclusions:**

The EvidencePoint system successfully demonstrated that a flexible, user-friendly platform for hosting CDS tools outside of a specific EHR is feasible. The forthcoming results of our outcomes analyses will demonstrate the adoption rate of EvidencePoint tools as well as the impact of behavioral economics “nudges” on the adoption rate. Due to the EHR-agnostic nature of EvidencePoint, the development process for additional forms of CDS will be simpler than traditional and cumbersome IT integration approaches and will benefit from the capabilities provided by the core system of EvidencePoint.

## Introduction

The practice of evidence-based medicine has well-established benefits, including improving patient satisfaction and health outcomes and reducing costs [1,2]. Through our work, we have demonstrated how clinical decision support (CDS) tools integrated into the electronic health record (EHR) assist providers in adopting evidence-based best practices and improving care [1,3,4]. However, although numerous studies on the benefits of EHR-integrated CDS tools have been published, widespread CDS adoption is limited [5-8].

In previous studies, we have demonstrated that effective clinical workflow integration is key to CDS adoption [3,9-14]. The more seamlessly a CDS tool is integrated into real-world EHR interfaces and procedures, the more likely it is to achieve high user-adoption rates. Achieving effective EHR integration for CDS tools, however, requires confronting technical challenges. CDS tools are typically built using an EHR’s proprietary development environment. As such, these tools are limited by EHR functionality, which is often rudimentary, frequently leading to the creation of tools that diverge from the ideal clinical implementation and disrupt clinical and digital workflows [15,16]. In addition, building a CDS tool “into” an EHR limits the ability to distribute the resulting CDS tool beyond the specific EHR it was developed to work with.

Our research had three implementation and dissemination goals: (1) to build the EvidencePoint platform and implement vendor-agnostic integrated CDS solutions for emergency room, inpatient, and ambulatory settings; (2) to perform comprehensive workflow analysis and usability testing with end-user clinicians to achieve optimal levels of tool usefulness, usability, and performance; and (3) to measure outcomes related to tool adoption rates and optimizations to clinical practice workflows.

## Methods

### Overview

To address CDS challenges, we developed EvidencePoint, a novel, cloud-based, EHR-agnostic CDS platform for the development and hosting of CDS tools. Here, we describe the development of EvidencePoint and the deployment of its initial CDS tools. The platform provides a library of CDS functionality for key clinical scenarios such as antibiotic prescribing, pulmonary embolism diagnosis, and pharmacological thromboprophylaxis. The platform includes an EHR trigger, clinical prediction rule (CPR), and order integration for each CDS tool. This modular functionality allows for the efficient expansion of additional tools. Clinical impact metrics are monitored through the system’s reporting dashboard.

Our team at Northwell’s Usability Lab was awarded an R18 grant through the Agency for Healthcare Research and Quality to begin the development of the EvidencePoint system in March 2019. This required collaboration among the usability lab members and several Northwell EHR and health information exchange (HIE) technical teams, along with an applications development team, a clinical documentation team, and a research innovations and informatics team. Over the course of the design and implementation process, considerations of scalability, portability, workflow, and user experience were paramount. All study activities were approved by the Northwell Health Institutional Review Board.

### Workflow Analysis

An important input to the integration of our CDS tools into EvidencePoint was to determine where in the clinical workflow our tools would appear and under which clinical circumstances. As an example, here we describe our process for determining where and how the Wells’ criteria CPR to detect pulmonary embolism CDS tool would be integrated into the emergency department’s (ED) clinical workflow. This methodology was also applied to the integration of all other CDS tools.

Initial workflow analysis for the Wells’ criteria CDS tool began with a task force meeting among the research team and key ED stakeholders, including frontline providers, the chairperson, administrators, and nurses. The meeting included semistructured questions with responses written into notes taken by a research coordinator and research administrator. The types of questions asked were the need for CDS in the ED, the current workflow of patients suspected of pulmonary embolism, where the tool would be needed for effective implementation, and how team members would interact with the tool. The focus was on system interactions and information flow.

After the workflow analysis meeting, research team members shadowed 4 different ED providers (frontline attending physician, resident physician, physician assistant, and primary ED nurse) to observe clinical workflow in real time. Each study team member was paired with 1 of the 4 ED providers. Study team members asked semistructured questions during the shadowing sessions and took notes on clinician responses. The observations, which ranged from 30 minutes to 1 hour, took place over several days in May 2019 in a tertiary care center’s ED.

### User Experience

The usability lab team’s previous work has demonstrated that a CDS tool’s usability is crucial to facilitating its adoption by providers [9-11,17,18]. Therefore, parallel to the development work on the underlying EvidencePoint software platform and workflow analysis, we made an effort to ensure the resulting CDS tools would be useful and usable for providers. Following meetings with key stakeholders, including attending physicians, residents, nurses, and physician assistants at each clinical site, the usability lab team started to map out user journeys and workflow analyses of the ED, inpatient hospital unit, and ambulatory clinical environments. Keeping in mind that the primary objective of the EvidencePoint system is to trigger relevant CPRs to the correct providers at the appropriate moments during the clinical decision-making workflow, the workflow analyses served as the preliminary road map for the ensuing usability testing process.

**Figure 1:**
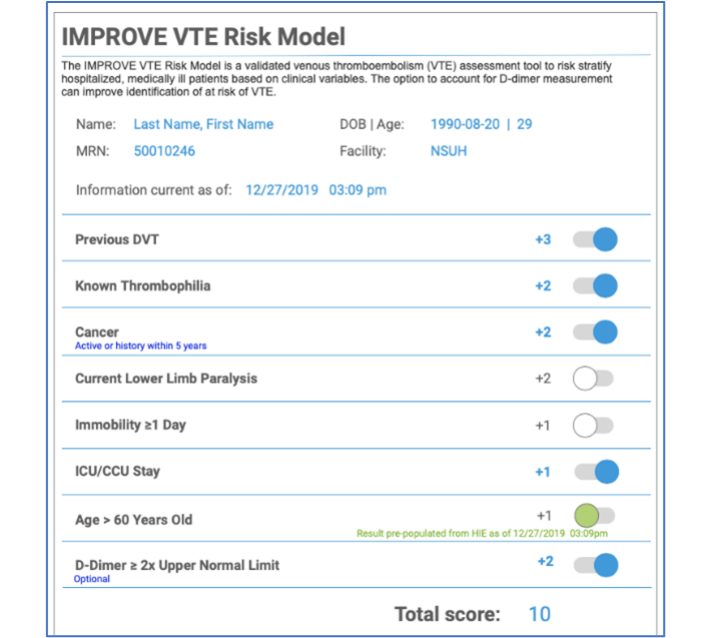
International Medical Prevention Registry on Venous Thromboembolism-D-Dimer clinical decision support application. CCU: critical care unit; DOB: date of birth; DVT: deep vein thrombosis; HIE: health information exchange; ICU: intensive care unit; MRN: medical record number; NSUH: North Shore University Hospital.

In addition to mapping user journeys and completing workflow analysis, the team also developed a series of interactive wireframe prototypes of potential user interfaces for each CDS tool that we were building. We performed initial rounds of rapid-cycle usability testing on these wireframes (Figure 1).

Our usability testing of the EvidencePoint platform and the integrated CDS tools is ongoing and will continue throughout the development process, including an assessment following the launch of each individual CDS tool to allow for an assessment of critical system analytics such as trigger rates. Our analyses will enable us to fine-tune the platform and the integrated CDS tools to maximize their impact on clinical outcomes while minimizing clinical workflow disruption.

### Technical Development of the EvidencePoint Platform

**Figure 2:**
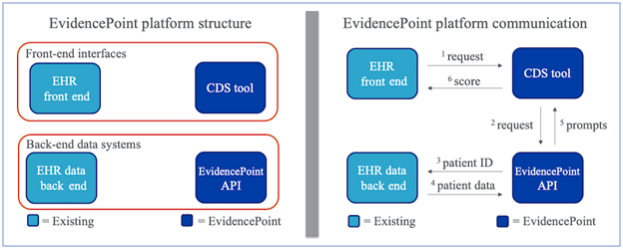
EvidencePoint platform structure (left) and communication scheme (right). API: application programming interface; CDS: clinical decision support; EHR: electronic health record.

The EvidencePoint platform is divided into 4 components that make up a front-end user interface and a back-end data exchange (Figure 2). The system was developed using standard web technologies, including HTML, CSS, and JavaScript. The front-end interface consists of the clinician-facing EHR and the integrated EvidencePoint CDS tool. The front-end EHR is responsible for launching and running the CDS tool, which is a web-based application that can be displayed within a native EHR window to appear as if it is an integrated part of the EHR clinical or digital workflow. The EvidencePoint CDS tools are hosted on a web server and calculate a predictive score based on CPR. CPRs are validated tools that quantify the individual contributions that components of history, physical, and laboratory results make toward a diagnosis, prognosis, or treatment response [19]. CPRs are prepopulated with patient data in the back-end data exchange, generating an automated CDS assessment.

The back-end data exchange consists of the EvidencePoint application programming interface (API) and the back-end EHRs, namely the HIE. Given the desired CDS tool, the EvidencePoint API translates patient health information (eg, test results and codes) from the HIE to the relevant assessment questions for prepopulation. Then, the API sends the prepopulated prompts and assessment-scoring scheme back to the CDS tool, where the doctor fills out the remainder of the assessment, corrects for errors, and calculates a score. The EvidencePoint software system thus bridges front-end, back-end, preexisting, and entirely bespoke software to bring CDS assessments to clinician workflows. The system currently supports several CDS applications, as described in the Results section.

To address scalability, each of the CDS tools—1 tool for each CPR—is configured on the EvidencePoint server with a text file. The text file specifies each assessment question, how the score is calculated, and the clinical codes relevant to assessment prepopulation. This modular design allows for a near “plug and play” development process for CDS administrators. As long as EHRs are configured to launch the EvidencePoint CDS tool, the system can provide clinicians with the desired CPR at the point of care, prepopulated with relevant patient data. The modular text-file configuration puts future CDS tools well within reach.

**Figure 3:**
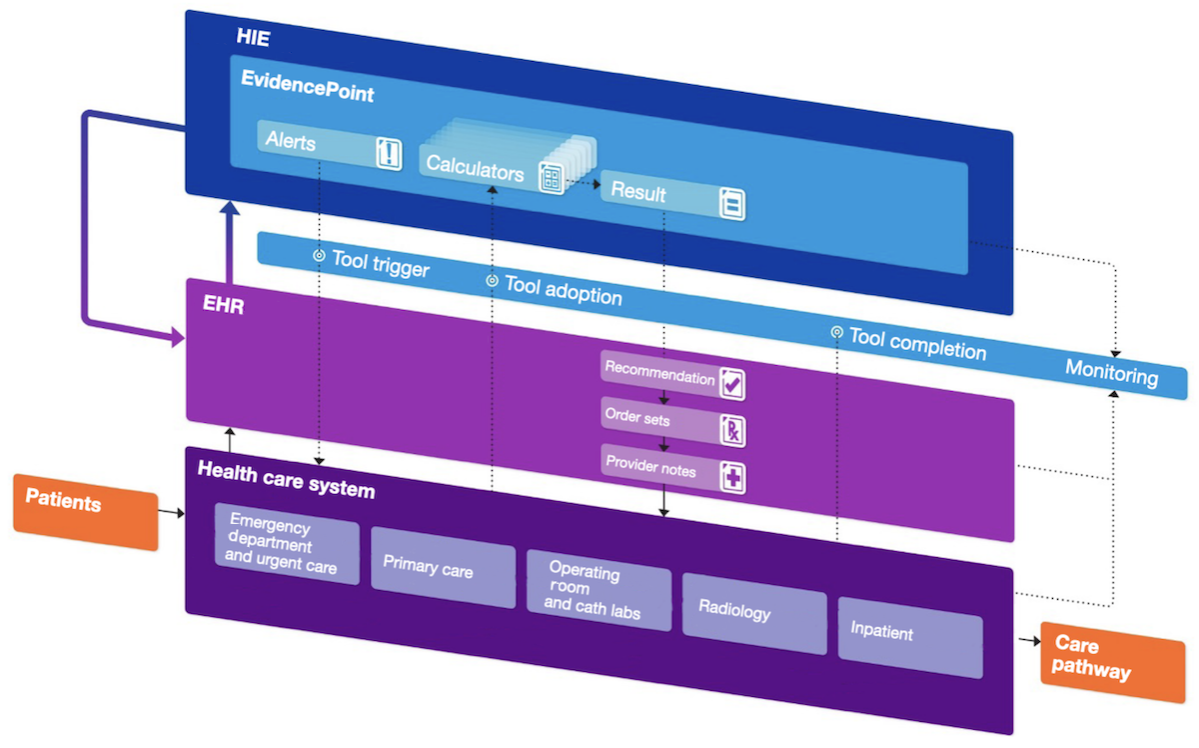
Schematics of the flow of data between the health care system, electronic health record (EHR), and health information exchange (HIE). Doctors launch an EvidencePoint clinical decision support tool from an EHR front-end workflow. The request includes the desired clinical prediction rule (eg, the Wells’ criteria) and the patient’s visit-specific ID. The clinical decision support tool forwards the request to the EvidencePoint application programming interface, which retrieves patient data, prepopulates evaluation answers, and sets the clinical prediction rule calculation logic. After calculating the patient score, the clinical decision support tool returns the score to the EHR front-end workflow.

To ensure portability to outside institutions and make widespread dissemination possible, the platform takes an EHR-agnostic approach. The CDS tools and EvidencePoint API run on separate servers from the Northwell EHRs and communicate through standard protocols. Thus, the platform can be configured to launch from any health system’s EHR based on that EHR’s particular data (Figure 3).

For example, from an end user perspective, the Wells’ criteria for pulmonary embolism CPR comprises several components [20-23], including “heart rate greater than 100” and “hemoptysis.” The EvidencePoint platform supports user-editable fields for each specific component of the CPR (eg, “heart rate greater than 100” and “hemoptysis”) and automatically calculates the associated point values for each (such as +1.5 for “heart rate greater than 100” and +1 for “hemoptysis”; Table 1). The user-editable field is pulled from the EHR and can be updated by the user if needed.

The text file also provides a mechanism to specify, on a component-by-component basis, the source for retrieving extant patient-specific data with which to prepopulate specific CPR components. The methods used to retrieve these data can include vendor-specific calls in addition to general data requests.

**Table 1:** The Wells’ score clinical prediction rule criteria.

Wells’ criteria (as seen by end user)	User-editable field	
Heart rate greater than 100	input:radio,Heart rate greater than 100,,,well	
		selected:Medical=1.5
		unselected:Medical=0
Hemoptysis	input:radio,Hemoptysis,,,wells	
		selected:Medical=1
		unselected:Medical=0

### CDS Tool Deployment

Following the development of the EvidencePoint platform and workflow analysis, we developed our first 3 CDS tools for the platform. We describe these CDS tools below.

#### Northwell COVID-19 Survival Tool

**Figure 4:**
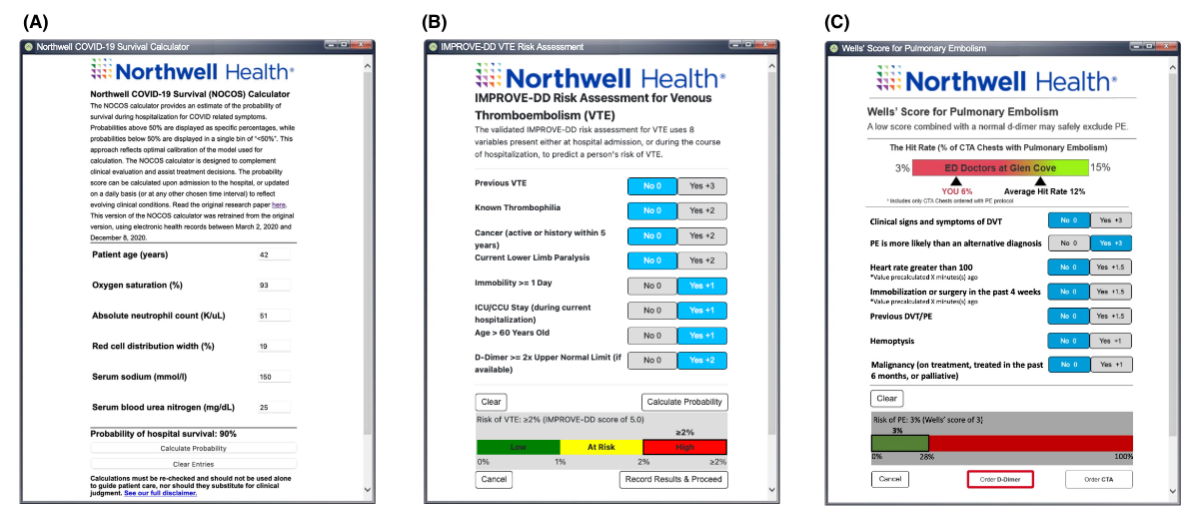
The Northwell COVID-19 Survival (NOCOS), the International Medical Prevention Registry on Venous Thromboembolism-D-Dimer (IMPROVE-DD), and the Wells’ Score clinical decision support applications. (A) The NOCOS tool for predicting a patient with COVID-19’s probability of surviving hospitalization. This noninterruptive tool is meant for use in an emergency department (ED) setting. (B) The IMPROVE-DD tool for predicting a patient’s risk of developing a venous thromboembolism. This interruptive tool is meant for use in an inpatient setting. (C) The Wells’ Score tool for assisting with the diagnosis of pulmonary embolism (PE). This interruptive tool is meant for use in the ED. CCU: critical care unit; CTA: computed tomography angiography; DVT: Deep Vein Thrombosis; EHR: electronic health record; ICU: intensive care unit.

One of the most important features of EvidencePoint is that it enables the rapid development, implementation, and dissemination of new CDS tools at the point of care, especially during times of urgent need. EvidencePoint’s debut came as the COVID-19 pandemic hit the New York City area, putting thousands of patients on ventilators, straining health care personnel, and stretching scarce medical supplies. Because the EvidencePoint platform enables the rapid development of CDS, Northwell Health’s research and software teams were able to respond quickly to the needs of the situation, developing and releasing the Northwell COVID-19 Survival (NOCOS) CPR [24], a tool that estimates a patient’s probability of survival during hospitalization. Following its success, the teams developed NOCOS 2.0, which integrated with the Allscripts Sunrise EHR to prepopulate patient information and return the NOCOS score back to the EHR for subsequent use. This was the first implementation of an EHR-integrated CDS tool running on the EvidencePoint platform (Figure 4A).

#### International Medical Prevention Registry on Venous Thromboembolism-D-Dimer Tool

Following the success of the NOCOS tool, our team released the International Medical Prevention Registry on Venous Thromboembolism-D-Dimer (IMPROVE-DD) tool (Figure 4B). This tool incorporates the IMPROVE-DD score for determining appropriate venous thromboembolism (VTE) prophylaxis. Hospital-acquired VTE is prevalent in the United States [25], with hospitalization as a significant risk factor in patients who are medically ill [26,27]. In addition, a medical patient’s risk of developing a VTE remains high up to 3 months post discharge [27]. The IMPROVE-DD scoring tool was developed based on the previous IMPROVE score [26], with the additional inclusion of the D-dimer blood test to better predict VTE risk [28] and was externally validated both in medical inpatients and inpatients with COVID-19 [27,29,30]. Therefore, due to the need for VTE risk assessment to determine appropriate prophylactic measures, especially during the pandemic, the IMPROVE-DD score was an excellent candidate for incorporation into the EvidencePoint platform. In comparison to NOCOS, IMPROVE-DD features had better integration with EHR workflows and more extensive usability testing. A total of 7 usability testing sessions were conducted for the IMPROVE-DD tool. The tool integrates seamlessly into 3 clinician workflows (ie, at admission history and physical, order entry, and discharge summary); prepopulates the assessment with relevant patient information; and returns a score that provides actionable patient recommendations that are tied to anticoagulant medication order entry to prevent VTE.

#### Wells’ Criteria Tool

From an implementation perspective, the EvidencePoint platform also allows us to add new functionality seamlessly to enhance our simpler previous CDS tools. Before EvidencePoint’s inception, we developed a version of the Wells’ criteria CDS tool [31]. A total of 7 usability testing sessions were completed for the Wells’ criteria tool. This tool was integrated into the EHR in a traditional format and has not been widely adopted by our intended end users (ED physicians and physician assistants) [31,32]. Including the Wells’ criteria CDS on the EvidencePoint platform (Figure 4C) will allow for rapid modifications of the tool that should enhance its usability, usefulness, and adoption. For example, the Wells’ criteria CDS will incorporate behavioral nudges aimed at encouraging clinicians to use the tool. The nudges will address areas that we have identified as barriers to the CDS tool’s use [32]. While we do not yet know which behavioral nudge will be most effective in terms of encouraging adoption of the Wells’ criteria CDS, our EvidencePoint platform will enable us to seamlessly switch among nudges so that we can study their impact.

### Ethical Considerations

The study was approved by Northwell Health’s institutional review board (protocol numbers 19-0045 and 18-0714) as minimal-risk research using observational data. Collected data were a part of routine clinical practice, and the requirement for informed consent was waived. Data were collected from the enterprise EHR (Sunrise Clinical Manager, Allscripts) reporting database. Adoption and usage data are aggregated, with no identifiable information included.

## Results

Shadowing sessions from the workflow analysis revealed several key feedback points. We addressed as many feedback points as possible in order to develop a tool that would be of ideal use to our end users. Key points noted during the meeting were the need to include an alert for the CDS in a manner that would not slow down the clinical workflow and the incorporation of the CDS early on during the clinical decision-making process. Additionally, usability testing provided valuable insight into aspects of the user experience that could be improved. As an example, the initial wireframe prototype for the IMPROVE-DD tool (Figure 1) presented users with the numerical point value of the CPR calculations the tool was performing. We learned from users that, rather than the absolute point value, it would be more beneficial to display the absolute risk percentage that users believe derives greater clinical meaning (Figure 4B).

The results below highlight the adoption of the CDS tools, the IMPROVE-DD, the Wells’ criteria, and the NOCOS, following development, usability testing, and implementation. The IMPROVE-DD CDS was used in 5249 patients at the 2 clinical intervention sites. The intervention group tool adoption was 77.8% (4083/5249 possible uses). For the NOCOS tool, which was designed to assist with triaging patients with COVID-19 for hospital admission in the event of constrained hospital resources, the worst-case resourcing scenario never materialized and triaging was never required. As a result, the NOCOS tool was not frequently used, though the EvidencePoint platform’s flexibility and customizability enabled the tool to be developed and deployed rapidly under the emergency conditions of the pandemic. Adoption rates for the Wells’ criteria tool will be reported in a future publication.

## Discussion

### Principal Findings

The EvidencePoint system successfully demonstrated that a flexible, user-friendly platform for hosting CDS tools outside of a specific EHR is feasible. The forthcoming results of our outcomes analyses will demonstrate the adoption rate of EvidencePoint tools as well as the impact of behavioral economics “nudges” on the adoption rate. Due to the EHR-agnostic nature of EvidencePoint, the development process for additional forms of CDS will be simpler than traditional and cumbersome IT integration approaches and will benefit from the capabilities provided by the core system of EvidencePoint.

With feasibility and proof-of-concept work completed, the IMPROVE-DD tool running on the EvidencePoint platform was released in production environments at 2 of Northwell’s largest tertiary hospitals in December 2020. In September 2021, the Wells’ criteria CDS tool was released in 2 tertiary hospitals and 1 community hospital. Once field-tested in these initial production environments, EvidencePoint will be capable of expanding to include additional forms of CDS (ie, dissemination and implementation of a child abuse CDS funded by Patient-Centered Outcomes Research Institute award No. DI-2017C1-6215) [33] and will be deployed at additional locations.

A recent study reviewed the design and implementation of CDS using 4 interoperability standards: Fast Healthcare Interoperability Resources (FHIR), Substitutable Medical Applications, Reusable Technologies (SMART), clinical quality language, and CDS Hooks [34]. A total of 44 studies were included in the review, of which 43 used FHIR, 22 used SMART, 2 used clinical quality language, and 8 used CDS Hooks. It was noted that evaluation of the technology was reported in a small number of studies, and many studies were in the design and piloting stages. Additional studies on CDS standards will be necessary to inform design decisions for further implementation and dissemination of the EvidencePoint platform moving forward.

Future work on EvidencePoint will include wider-scale dissemination, enabling the platform to be used beyond the Northwell environment. We will enhance EvidencePoint to fully support evolving development standards, such as SMART on FHIR, and provide operators of hospitals and ambulatory sites with a novel tool kit of usability-tested, workflow-integrated functionality directly aimed at bringing sophisticated evidence-based medicine to the point of care.

Due to the EHR-agnostic nature of EvidencePoint, the development process for these additional forms of CDS will be simpler than traditional and cumbersome IT integration approaches and will benefit from the capabilities provided by the core system. This new functionality will be available to multiple EHRs from various vendors since it will reside in a cloud system as opposed to being built directly into the EHR. As evidenced in the previously detailed NOCOS example, we will be able to quickly modify individual CDS tools to meet the needs of our end users and optimize our tools’ adoption and use. As noted, behavioral nudges will be activated to address barriers to CDS uptake and usage. By providing a library of evidence-based CDS tools that can be integrated directly into the flow of care while also being easy to disseminate to multiple locations on an EHR-agnostics basis, EvidencePoint will help to reduce the “evidence gap” that has traditionally made it difficult to use evidence at the point of care.

### Limitations

A few limitations are noted as the results of the ongoing outcomes study are forthcoming. Future publications will discuss ordering behavior, adoption and acceptance by the provider, and the overall effectiveness of the CDS tools in clinical outcomes (ie, decreased hospital-acquired VTE and decreases in unnecessary diagnostic testing for pulmonary embolism based on patient risk stratification). Currently, the IMPROVE-DD tool has been deployed in 4 clinical sites in a clustered randomized controlled trial as part of an impact analysis, and improved outcomes will be reviewed and forthcoming. In addition, while we have attempted to address all of our end-user feedback, the technological limitations of our EHR prevent us from making some of the changes that would further enhance the user experience with the system.

Although the study was limited to the Northwell Health System, it included the implementation of the EvidencePoint platform and CDS tools at academic tertiary centers and community-based hospitals. Given the size of the health system as well as the vast range of differentiated demographics among the patient population during one of the greatest health crises in recent history, this constraint alleviated much of the team’s concern regarding further implementation and dissemination of the system.

### Conclusions

Due to the EHR-agnostic nature of EvidencePoint, the development process for additional forms of CDS will be simpler than traditional and cumbersome IT integration approaches and will benefit from the capabilities provided by the core system of EvidencePoint.

## Abbreviations

API: application programming interface
CDS: clinical decision support
CPR: clinical prediction rule
ED: emergency department
EHR: electronic health record
FHIR: Fast Healthcare Interoperability Resources
HIE: health information exchange
IMPROVE-DD: International Medical Prevention Registry on Venous Thromboembolism-D-Dimer
NOCOS: Northwell COVID-19 Survival
SMART: Substitutable Medical Applications, Reusable Technologies
VTE: venous thromboembolism

## Data Availability

The data sets generated and analyzed during this study are available from the corresponding author on reasonable request.
